# The effects of fine and coarse particulate matter on lung function among the elderly

**DOI:** 10.1038/s41598-019-51307-5

**Published:** 2019-10-15

**Authors:** Chi-Hsien Chen, Chih-Da Wu, Hung-Che Chiang, Dachen Chu, Kang-Yun Lee, Wen-Yi Lin, Jih-I Yeh, Kun-Wei Tsai, Yue-Liang Leon Guo

**Affiliations:** 1 Department of Environmental and Occupational Medicine, National Taiwan University Hospital Hsin-Chu Branch, Hsinchu City, Taiwan; 2 Department of Environmental and Occupational Medicine, National Taiwan University (NTU) College of medicine and NTU Hospital, Taipei City, Taiwan; 3 Department of Geomatics, National Cheng Kung University, Tainan City, Taiwan; 4 National Institute of Environmental Health Sciences, National Health Research Institutes, Miaoli County, Taiwan; 5 School of Medicine, National Yang-Ming University, Taipei City, Taiwan; 6 Institute of Public Health and Community Medicine Research Center, National Yang-Ming University, Taipei City, Taiwan; 7 Department of Health Care Management, National Taipei University of Nursing and Health Sciences, Taipei City, Taiwan; 8 Department of Neurosurgery, Taipei City Hospital, Taipei City, Taiwan; 9 Division of Pulmonary Medicine, Department of Internal Medicine, Shuang Ho Hospital, Taipei Medical University, New Taipei City, Taiwan; 10 Department of Internal Medicine, School of Medicine, College of Medicine, Taipei Medical University, Taipei City, Taiwan; 11 Department of Occupational Medicine, Health Management Center, Kaohsiung Municipal Siaogang Hospital, Kaohsiung Medical University, Kaohsiung City, Taiwan; 12 Department of Family Medicine, Hualien Tzu-Chi General Hospital, Hualien, Taiwan; 13 Division of Geriatrics, Dalin Tzu-Chi Hospital, Buddhist Tzu Chi Medical Foundation, Chia-Yi, Taiwan; 14 Institute of Environmental and Occupational Health Sciences, National Taiwan University, Taipei City, Taiwan

**Keywords:** Geriatrics, Epidemiology, Epidemiology, Risk factors

## Abstract

Impaired lung function is associated with morbidity and mortality in the elderly. However, there is a paucity of data regarding the long-term effects of particulate matter (PM) on lung function among the elderly. This study evaluated the exposure-response relationship between ambient PM and different lung function indices among the elderly in Taiwan. A cross-sectional survey of individuals aged ≥65 years was conducted in Taiwan from October 2015 to September 2016. Those who attended the annual health examination for the elderly in five hospitals of varying background PM concentrations were enrolled. The long-term (2015 annual mean concentration) exposure to air pollution was estimated by the Kriging method at the residence of each subject. The association between ambient PM exposure and lung function was evaluated by linear regression modeling, with adjustments for age, sex, height, weight, educational attainment, presence of asthma or chronic obstructive pulmonary disease, smoking status, season, and co-pollutants. There were 1241 subjects (mean age, 70.5 years). The mean residential PM_2.5_ and PM_2.5–10_ in 2015 was 26.02 and 18.01 μg/m^3^, respectively. After adjustments for confounders and co-pollutants, the FVC decrease was best associated with fine particles (PM_2.5_), whereas the FEV_1_, FEF_25–75%_, FEF_25%_ and FEF_50%_ decreases were best associated with coarse particles (PM_2.5–10_). An IQR (10 μg/m^3^) increase in PM_2.5_ decreased FVC by 106.38 ml (4.47%), while an IQR (7.29 μg/m^3^) increase in PM_2.5–10_ decreased FEV_1_ and FEF_25–75%_ by 91.23 ml (4.85%) and 104.44 ml/s (5.58%), respectively. Among the Taiwanese elderly, long-term PM_2.5_ exposure mainly decreases the vital capacity of lung function. Moreover, PM_2.5–10_ has a stronger negative effect on the function of conductive airways than PM_2.5_.

## Introduction

Lung function is a known predictor of mortality risk. Large follow-up studies in adults have provided evidences that lower baseline force vital capacity (FVC) and forced expiratory volume in one second (FEV_1_) are associated with higher all-cause mortality risk^1,2^. While there is substantial evidence on the short- and long-term effects of ambient air pollution on lung function in children and adults^3,4^, few studies have focused on the long-term effects of air pollution on lung function among the elderly, a vulnerable population. Previous research conducted in low polluted areas (10.8 μg/m^3^ of annual average PM_2.5_) reveals a exposure-response relationship between long-term PM_2.5_ exposure and lung function decrement in the middle- to older-aged pollution, with 18.7 ml (0.45%) and 13.5 ml (0.42%) decreases in FVC and FEV_1_, respectively, for every 2 μg/m^3^ increase in PM_2.5_^5^. However, there is paucity of information in highly polluted areas.

In 2015, the median of the annual average PM_2.5_ in Taiwan was approximately 22.5 μg/m^3^, which was more than twice that of the regulated levels proposed by the World Health Organization (10 μg/m^3^). A previous study in Taiwan showed stronger effects of sub-chronic (two-month period) exposure to ambient fine particles and ozone on lung function in schoolchildren compared to that of acute exposure (lag 1 day). A 10 μg/m^3^ increase in sub-chronic PM_2.5_ exposure (Q1–Q3 at 32–44 μg/m^3^) was also associated with a 3.3% and 3.1% reduction in FVC and FEV1, respectively, which in turn was 40% and 48% larger, respectively, than the effects found in a relatively less polluted area (Q1–Q3 at 10–17 μg/m^3^)^6^. Whether or not the elderly in Taiwan have a similar response to ambient air pollution as children do is not known. The observed exposure-response relationship of long-term air pollution on lung function in the elderly may be used to predict mortality burden and may be informative for policy makers in areas with relatively high ambient air pollution.

Inhalable particulate matter includes fine and coarse particles with aerodynamic diameter ≤2.5 μm (PM_2.5_) and 2.5–10 μm (PM_2.5–10_), respectively. The main source of PM_2.5_ is the combustion of fossil fuel and the high-temperature industrial process, whereas that of PM_2.5–10_ includes mechanical disruption of various materials and microbial fragments^7^. According to previous deposition models^8–10^, particles with aerodynamic diameter >10 μm deposit approximately 100% in the human nose or extra-thoracic airway during rest and light exercise, while inhaled coarse particles of 3–6 μm can reach and deposit in the lower respiratory tract. Moreover, PM_2.5_ can penetrate deep into the alveolar region, but PM_2.5–10_ deposits mainly in the tracheo-bronchial airways.

Given the variations in source, composition, and airway deposition between fine and coarse PM, there may be different patterns of health effects. An integrated science assessment by the United States Environmental Protection Agency considers the respiratory effects of long-term PM_2.5_ exposure as “likely to be causal”, but only “suggestive” for PM_2.5–10_ due to the lack of epidemiologic information^7^. Identifying the different health effects of fine and coarse PM may provide the foundation for source controlling policy and disease prevention.

## Materials and Methods

### Study design and subjects

We conducted a cross-sectional survey from October 2015 to September 2016 on the elderly (age ≥65 years) who lived in five areas in Taiwan (including Taipei city, New Taipei city, Hualien county, Chiayi county, and Kaohsiung city) with varying background PM concentration. The elders were consented to participate in this study during their annual geriatric health examination. To resemble general elderly population, we excluded those with malignancy and difficulties in mobility and general communication. The study was approved by the institution review board of the National Health Research Institutes (EC 1040508-E-R2) and was conducted in accordance with relevant guidelines and regulation. Each participant has signed the informed consent.

### Questionnaire

We designed a standard questionnaire to collect information including personal habits (such as cigarette smoking, alcohol consumption, etc.), medical conditions (underlying diseases and treatment received), and educational attainment. In order to overcome some difficulties with questionnaire completion (such as visual impairment, reading ability, etc.), five well-trained interviewers conducted the interviews.

### Measurement of pulmonary function

Lung function was measured by five well-trained technicians using spirometer (Otthon Mobile Handheld Spirometer, THOR Asian Pacific), and according to the standard of the American Thoracic Society. Each spirometer was calibrated monthly using 3 L flow-volume syringes. The elders underwent spirometry in the sitting position, in doors, and in the morning. The forced vital capacity (FVC), forced expiratory volume in 1 second (FEV_1_), FEV_1_/FVC ratio, and forced expiratory flow rates at different lung volumes (including FEF_25%_, FEF_50%_, FEF_75%_, and FEF_25–75%_) were measured. We collected at least three acceptable spirograms per subject. An acceptable spirogram was defined as good start of blowing without hesitation, smooth blowing curve without artifacts, and at least 6 seconds of expiratory duration, or with a plateau >1 second in the end expiration in the volume-time curve. A maximum of 8 blows were allowed for each lung function test.

We only included the tests where the differences between the two largest FVC and FEV_1_ were both within 150 ml. We also measured the body height and weight at the time of spirometry for adjustment.

### Assessment of air pollution exposure

Each subject’s exposure to ambient air pollution was estimated based on his or her residential site. We also retrieved the data of hourly levels of pollutants including PM_2.5_, PM_10_, nitrogen dioxide (NO_2_), carbon monoxide (CO), ozone (O_3_), and sulphur dioxide (SO_2_), from 73 Taiwan Environmental Protection Administrion (EPA) monitoring stations, to calculate the one-year average concentration of air pollution in 2015. The kriging method developed by Liao *et al*.^11^ were used to estimate the long- term residential exposure of air pollution.

Spatial estimation of pollution concentration was done by using the ArcView GIS(version 9.3) program. 10-Fold cross-validation was applied to verify the reliability of Kriging estimation. In brief, 90% of the air quality monitoring stations (training dataset) were randomly selected for Kriging interpolation and the remaining 10% used as validation dataset^12^. Particulate matter with aerodynamic diameter of 2.5–10 μm (PM_2.5–10_) was derived by subtracting PM_2.5_ from PM_10_.

### Statistical analysis

We calculated the Pearson’s correlation coefficient between each air pollutant and examined the relationship between the air pollutants and lung function by multiple linear regression, using the JMP software version 5.0 (SAS Institute, Gary, NC, USA). To evaluate the long-term effect of air pollution on lung function, only subjects living in the current residential site for more than one year were included in the statistical analysis.

The association between each air pollutant with respiratory function indices was first examined. From the correlation between air pollutants, the two-pollutant model was used to identify the most significant pollutant. All of the models were adjusted for a set of variables chosen based on previous biologic and epidemiologic knowledge. These variables included age, sex, height, weight, smoking status (past or current smoker and pack-year), diagnosed asthma or chronic obstructive pulmonary disease (COPD), educational attainment, and season of respiratory function test. An investigation of a potential interaction between the effects of PM_2.5_ and PM_2.5–10_ on lung function was done by calculating the p value of interaction term. A subgroup analysis excluding subjects with physician-diagnosed asthma and COPD was also performed to confirm the association between air pollution and lung function. Statistical significance was set at *p* < 0.05.

## Results

### The characteristics of study subjects

**Figure 1:**
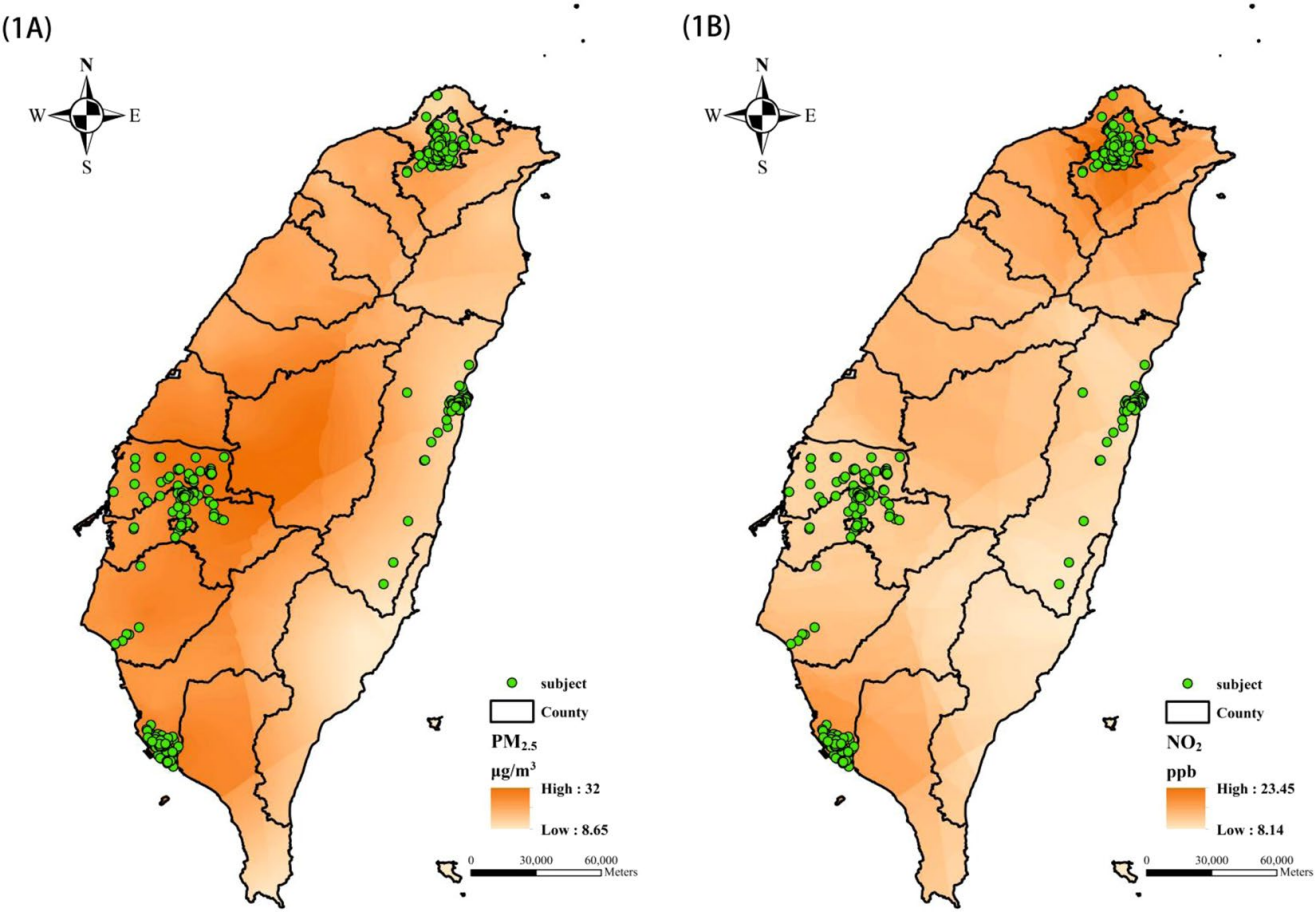
The residential locations of the study subjects (n = 1241) and the Kriging estimation of ambient PM_2.5_ and NO_2_ for the year 2015. **(A)** PM_2.5_. **(B)** NO_2_.

The residential locations of the participants were shown in Fig. 1, while their demographics and respiratory function indices were summarized in Table 1. There were 1241 elderly in this study (mean age, 70.5 years; male-to-female ratio, 0.73). Their mean BMI was 24.6 kg/m^2^ and 42.8% had a BMI >25 kg/m^2^. Among them, 8.6% were current smokers and 11.3% were former smokers, with a median time from quitting smoking of 18.5 years. The mean cumulative pack-years of current and former smokers were 30.6 and 18.3, respectively. The prevalence of diagnosed asthma with airway symptoms in the past year and COPD were 2.0% and 1.9%, respectively.

**Table 1:** Characteristics of the elderly who participated in the Taiwan Aging Cohort Study, 2015–2016 (n = 1241).

	Mean (SD) or %	
	Male	Female
Number	522	719
**Demographics**		
Age, yr	70.91 (4.38)	70.16 (4.07)
Body height, cm	163.22 (6.18)	154.24 (6.69)
Body weight, kg	66.19 (9.40)	58.13 (9.77)
Body mass index	24.83 (3.11)	24.39 (3.48)
**Education level**		
Low(primary school or less)	38.12	48.82
Medium (middle or high school or equivalent)	45.02	44.65
High (university degree or more)	16.86	6.54
**Smoking status, %**		
Never	61.30	93.74
Current	14.75	4.17
Former	23.95	2.09
Pack-years*	23.33 (16.44)	25.04 (18.35)
Physician diagnosed asthma, %	2.87	1.39
Physician diagnosed COPD, %	2.49	1.39
**Lung function indices**		
FVC, ml	2872.89 (560.99)	2026.23 (417.74)
FEV1, ml	2235.00 (482.24)	1622.52 (336.17)
FEF_25~75%_, ml/s	2138.03 (849.44)	1675.52 (636.93)
FEF_25%_, ml/s	5054.14 (1680.46)	3761.57 (1098.84)
FEF_50%_, ml/s	2869.41 (1118.19)	2248.80 (831.12)
FEF75%, ml/s	811.53 (413.43)	645.83 (332.55)
FEV_1_/FVC	0.78 (0.08)	0.80 (0.07)
**Season of lung function test, %**		
Spring (March-May)	52.11	49.65
Summer (June-August)	43.10	45.20
Fall (September-November)	1.15	1.53
Winter (December-February)	3.64	3.62

*Among current or former smokers.

### The distribution of air pollution at residential sites

Distributions of the 2015 annual concentrations of six air pollutants were shown in Table 2. The mean residential PM_2.5_ was 26 μg/m^3^, which exceeded the National Ambient Air Quality Standard of Taiwan of 15 μg/m^3^. However, the mean NO_2_, CO, O_3_, and SO_2_, concentrations were below the national regulated levels. The PM_2.5_ and NO_2_ inter-quartile ranges (IQR) were wide, 10 μg/m^3^ and 9.9 ppb compared to their mean concentrations of 26 μg/m^3^ and 16.5 ppb, respectively. Moreover, PM_2.5_ level was highly correlated with PM_2.5–10_ (r = 0.82) and SO_2_ (r = 0.81) levels, whereas PM_2.5–10_ level was moderately correlated with SO_2_ (r = 0.63) (Table 3). The 10-fold cross-validated R^2^ values of the Kriging estimation of PM_2.5_, PM_10_, NO_2_, CO, O_3_, and SO_2_ were 0.61, 0.50, 0.63, 0.28, 0.20 and 0.61, respectively.

**Table 2:** Distributions of air pollution exposure in residences of the elderly (n = 1241).

	Mean	Median	IQR	Minimum	Maximum
**Exposures in 2015**					
PM_2.5_, μg/m^3^	26.02	24.53	10.00	17.05	35.33
PM_2.5–10_, μg/m^3^	18.01	16.54	7.29	10.74	30.21
NO_2_, ppb	16.54	15.79	9.93	8.63	21.62
CO, ppm	0.55	0.45	0.33	0.34	0.73
O_3_, ppb	27.03	27.10	1.68	25.80	30.40
SO_2_, ppb	3.24	3.21	0.23	1.76	4.59

Definition of abbreviations: IQR, interquartile range; PM_2.5_, particulate matter with aerodynamic diameter of 2.5 μm; PM_2.5–10_, particulate matter with aerodynamic diameter of 2.5–10 μm; NO_2_, nitrogen dioxide; CO, carbon monoxide; O_3_, ozone; SO_2_, sulphur dioxide.

**Table 3:** Correlations of air pollutants for the year 2015 in residences of the elderly (n = 1241).

	PM_2.5_	PM_2.5–10_	NO_2_	CO	O_3_	SO_2_
PM_2.5_	1	0.820**	0.001	−0.224**	0.109*	0.807**
PM_2.5–10_		1	−0.272**	−0.473**	0.364**	0.629**
NO_2_			1	0.967**	−0.879**	0.363**
CO				1	−0.862**	0.117**
O_3_					1	−0.249**
SO_2_						1

Abbreviations: PM_2.5_, particulate matter with aerodynamic diameter of 2.5 μm; PM_2.5–10_, particulate matter with aerodynamic diameter of 2.5–10 μm; NO_2_, nitrogen dioxide; CO, carbon monoxide; O_3_, ozone; SO_2_, sulphur dioxide.

*p < 0.001; **p < 0.0001.

### The effect of air pollution on lung function, single-pollutant model

Estimations of the association between each pollutant and respiratory function indices were shown in Table 4. Both PM_2.5_ and PM_2.5–10_ were significantly associated with decrements in FVC, FEV_1_, and FEF_25%_. On the other hand, PM_2.5–10_ had negative associations with FEF_25–75%_ and FEF_50%_, while SO_2_ had relatively weak associations with FVC, FEV_1,_ and FEF_25%_. An IQR change in PM_2.5_ was associated with 106.38 ml (4.47%) decrease in FVC and 73.30 ml (3.90%) decrease in FEV_1_. An IQR change in PM_2.5–10_ was associated with 101.22 ml (4.25%), 91.23 ml (4.85%), and 104.44 ml/s (5.58%) decrease in FVC, FEV_1_, and FEF_25–75%_, respectively. The association between air pollution and lung function indices remained in the subgroup without physician-diagnosed asthma and COPD (Supplementary Table S1).

**Table 4:** Association between each ambient air pollutant and lung function indices, in a single-pollutant model (n = 1241).

	FVC	FEV_1_	FEV_1_/FVC
PM_2.5_	−106.38 (23.01)****	−73.30 (18.97)***	0.005 (0.004)
PM_2.5–10_	−101.22 (25.84)****	−91.23 (21.23)****	−0.003 (0.004)
NO_2_	0.54 (28.93)	9.74 (23.80)	0.003 (0.005)
CO	28.58 (28.55)	28.48 (23.48)	0.001 (0.005)
O_3_	−15.00 (26.17)	−18.40 (21.52)	−0.003 (0.004)
SO_2_	−14.60 (4.18)***	−8.68 (3.44)*	0.001 (0.001)
	**FEF**_**25–75%**_	**FEF**_**25%**_	**FEF**_**50%**_
PM_2.5_	−26.75 (36.57)	−420.99 (67.33)****	−87.33 (48.05)
PM_2.5–10_	−104.44 (40.88)*	−495.67 (75.33)****	−168.51 (53.71)**
NO_2_	28.65 (45.60)	74.71 (85.25)	0.22 (60.00)
CO	31.08 (45.01)	217.89 (83.94)**	17.85 (59.22)
O_3_	−43.32 (41.23)	−99.83 (77.08)	−43.88 (54.25)
SO_2_	2.21 (6.61)	−74.47 (12.18)****	−7.17 (8.70)

^*^p < 005; **p < 0.01; ***p < 0.001; ****p < 0.0001.

The models were adjusted by age, sex, body height, body weight, diagnosed asthma and COPD, educational attainment, smoking status (e.g. current or past smoker, cumulative pack-year of smoking), and season of lung function test.

The regression coefficients and standard errors were estimated for every interquartile range increase in each pollutant, 10 μg/m^3^ for PM_2.5_, 7.29 μg/m^3^ for PM_2.5–10_, 9.93 ppb for NO_2_, 0.33 ppm for CO, 1.68 ppb for O_3_, and 0.23 ppb for SO_2_.

### Two-pollutant model to clarify the most hazardous air pollutant

The two-pollutant model was used to adjust for the potential confounding effects of co-pollutants (Table 5). The effect of PM_2.5_ on FVC remained consistently significant, as well as the effects of PM_2.5–10_ on FEV_1_, FEF_25–70%_, FEF_25%_, and FEF_50%_. The effect of SO_2_ on respiratory function was insignificant after adjusting for particulate matters. There was no significant synergistic interaction between the effect of PM_2.5_ and PM_2.5–10_ on lung function (Supplementary Table S2). In the subgroup analysis of subjects without obstructive lung diseases, the effect of PM_2.5_ on FVC, and PM_2.5–10_ on FEF_25–70%_, FEF_25%_, and FEF_50%_ were similar after adjusting for co-pollutants (Supplementary Table S3).

**Table 5:** The association between each ambient particulate air pollutant and lung function indices, in a two-pollutant model (n = 1241).

	FVC	FEV_1_	FEV_1_/FVC	FEF_25–75%_	FEF_25%_	FEF_50%_
**PM**_**2.5**_						
with PM_2.5–10_	−94.58*	−22.89	0.02**	131.70*	−187.05	90.49
with NO_2_	−106.63****	−73.82***	0.005	−27.87	−424.67****	−87.53
with CO	−105.41****	−71.43***	0.005	−23.24	−402.79****	−87.73
with O_3_	−105.99****	−72.47***	0.005	−23.89	−416.61****	−84.81
with SO_2_	−113.84**	−94.69**	0.001	−97.73	−254.33*	−150.11
**PM**_**2.5–10**_						
with PM_2.5_	−16.57	−70.74*	−0.02**	−222.33**	−328.24**	−249.50**
with NO_2_	−107.47****	−90.71****	−0.003	−104.36*	−509.51****	−179.07**
with CO	−110.61***	−98.37****	−0.004	−113.35*	−503.95****	−198.49***
with O_3_	−109.21***	−96.51****	−0003	−101.77*	−524.44****	−174.39**
with SO_2_	−73.37*	−91.25***	−0.011*	−172.55***	−342.63***	−218.25**
**SO**_**2**_						
with PM_2.5_	1.70	4.88	0.001	16.21	−38.05	14.33
with PM_2.5–10_	−7.62	0.005	0.002*	18.63*	−41.86**	13.61
with NO_2_	−17.89***	−11.34**	0.001	0.53	−96.66****	−8.77
with CO	−16.09***	−9.92**	0.001	1.35	−84.30****	−8.02
with O_3_	−16.70***	−10.41**	0.001	0.22	−86.35****	−10.05

^*^p < 005; **p < 0.01; ***p < 0.001; ****p < 0.0001.

The models were adjusted for age, sex, body height, body weight, diagnosed asthma and COPD, educational attainment, smoking status (e.g. current or past smoker, cumulative pack-year of smoking), season of lung function test, and co-pollutants.

The regression coefficients were estimated for every interquartile range increase in each pollutant.

## Discussion

The results show significant negative effects of long-term exposure to both ambient PM_2.5_ and PM_2.5–10_ on lung function among Taiwanese elderly living in four geographic areas. There is a wide distribution of PM concentrations and different effects of PM_2.5_ and PM_2.5–10_ on lung function parameters when using the two-pollutant model. Long-term PM_2.5_ exposure mainly decreases the vital capacity of lung function, whereas PM_2.5–10_ has a stronger negative effect on airway function. Overall, for every IQR (10 μg/m^3^) increase in PM_2.5_, FVC is reduced by 106.38 ml (4.47%) and for every IQR (7.29 μg/m^3^) increase in PM_2.5–10_, FEV_1_ and FEF_25–75%_ are reduced by 91.23 ml (4.85%) and 104.44 ml/s (5.58%), respectively.

The exposure-response relationship of PM_2.5_ on lung function is larger than in previous research conducted in the area of a relatively lower level of air pollution. The results here reveal that each 10 μg/m^3^ increase in PM_2.5_ reduces FVC and FEV1 by 106.38 ml (4.47%) and 73.30 ml (3.90%), respectively. Adar *et al*.^13^ reported the association between PM_2.5_ and lung function in the Multi-Ethnic Study of Atherosclerosis (MESA). The MESA recruited middle-aged adults and elderly (45~84 years of age) in six U.S. states. The mean 1-year PM_2.5_ exposure of participants was approximately 14 μg/m^3^. Their results revealed that every 10 μg/m^3^ increase in PM_2.5_ reduced FVC and FEV_1_ by 108 ml (3.3%) and 48 ml (2%), respectively. The larger effect size in relatively more highly polluted area underscores the need for an active policy to cut ambient PM_2.5_ in highly polluted areas.

The current study reveals a stronger effect of PM_2.5–10_ on lung function parameters related to conductive airways than PM_2.5_. Prior experiments on respiratory drugs have disclosed much higher deposition rates of coarse particles on extrathoracic and upper bronchial regions of respiratory tract than fine particles^14^. As for the lower bronchial region, there seems to be similar preference for the deposition of fine and coarse particles. Our study showed the preferential effect of PM_2.5–10_ on FEV_1_ and MMEF. Upon lung function parameters, FEV_1_ and FEV_1_/FVC ratio generally represent larger airway function and MMEF to smaller airway function. In other words, the observed effect of PM_2.5–10_ on FEV_1_ in this study echoes previous evidences disclosing its dominant deposition in larger airways, but its effect on mid-expiratory flow also suggests an impact of ambient PM_2.5–10_ down to lower bronchial region.

Although fine PM has been considered highly dangerous, evidence shows that ambient coarse PM also causes several health hazards, e.g. increased blood pressure^15^, heart rate variability^16^, respiratory morbidity and mortality^17^, emergency visit for asthma^18^, and neural biomarkers^19^. Some studies also show the lung function effects of PM_10_ or PM_2.5_^20^, but information remains very limited for PM_2.5–10_^7^. The multi-center European meta-analysis, ESCAPE, has found that a 10 μg/m^3^ increase in long-term exposure to PM_10_, but not in PM_2.5_ and PM_2.5–10_, is associated with approximately 1.4% and 1.5% decreases in FEV_1_ and FVC, respectively^21^. In a cross-sectional study in southern China, areas with an average PM_2.5_ of 23–75 μg/m^3^ demonstrate a stronger PM_2.5_ effect on FEV_1_ and FVC than PM_10_^22^, suggesting that fine PM has the main effect on lung function. The inconsistent results may be due to the varying compositions of ambient PM, weather conditions, and co-pollutants in different geographic areas. Results of the present study finding may trigger more study interests regarding the health effects of PM_2.5–10_. Given the differences in sources and formation mechanisms between fine and coarse PM, various environmental strategies or policies should be integrated to minimize overall PM-related health problems.

In this study, there are different patterns of PM_2.5_-and PM_2.5–10_-related lung function impairments. In the single-pollutant model, there are non-significant positive and negative associations for PM_2.5_ and PM_2.5–10_, respectively, with FEV_1_/FVC ratio, suggesting the restrictive effect of PM_2.5_ and the obstructive effect of PM_2.5–10_. In the two-pollutant model, the consistent effects of PM_2.5_ on FVC and PM_2.5–10_ on FEV_1_ and FEF_25–75%_ further suggest the anatomic tropism of various sized PM. Whether ambient PM restricts or obstructs lung function remains controversial in epidemiologic research.

The Normative Aging Study of elderly men reveals that the effect of long-term black carbon exposure is stronger for FEV_1_ than for FVC^23^, suggesting an obstructive pattern. A recent study in southern China has demonstrated a significant obstructive effect of long-term PM_2.5_ exposure^22^, while the cross-sectional analysis of the SAPALDIA study shows a stronger effect of PM_10_ on FVC than on FEV_1_^24^. This points to a restrictive effect. The ESCAPE meta-analysis has observed a significantly stronger effect of PM_10_ on FVC than FEV_1_^21^, also suggesting a restrictive pattern. In the same study, further looking into the non-significant results of PM_2.5_, PM_2.5_ absorbance (an indicator of black carbons), and PM_2.5–10_, there is a tendency for a restrictive effect of PM_2.5_ and obstructive effects of PM_2.5_ absorbance and PM_2.5–10_. More studies are needed to determine whether the specific size or composition of PM can lead to different lung function impairments.

Overall, this study does not show any statistically significant obstructive or restrictive effect of PM by FEV_1_/FVC ratio, but rather size-specific effects on parameters of vital capacity (FVC) or airway function (FEV_1_ and FEF_25–75%_). A recent nationwide survey of schoolchildren aged 6–15 years in Taiwan also reveals similar findings: a non-significant trend of a positive association between FEV_1_/FVC ratio and PM_2.5_, especially in children aged 11–15 years, and a negative association between FEV_1_/FVC ratio and PM_2.5–10_^6^. Based on previous deposition models in humans^7–10^, there is no clear boundary for the deposition of various sized PM on the tracheobronchial airway or alveolar region. During light exercise, any sized inhalable PM can deposit in every part of the respiratory system, with a higher proportion of coarse PM depositing in the tracheobronchial region than in the alveolar region, whereas fine PM is deposited mostly in the alveolar region. Thus, PM of various sizes may theoretically have varying proportional effects on each part of the intra-thoracic airways. However, the FEV_1_/FVC ratio mainly measures the airway obstruction in the larger airways. Additional examinations, including total lung capacity and lung diffusing capacity, may improve the assessment of PM_2.5_ effects on alveolar regions.

This study has some strengths. First, the relatively lengthy residential duration (>5 years in 92% of the participants) in our study population may improve the accuracy of long-term exposure assessments. Second, the elder population tends to spend most time at home and has smaller daily activity catchment area. This also improves our assessment accuracy, compared to younger and working population. Third, the high spatial heterogeneity and wide range of PM exposure level in our study population allowed the associations of PM and lung function to be well documented (Table 4). Fourth, the study was conducted in areas with high density of air monitoring stations. This reduces the misclassification from Kriging interpolation. Lastly, standardized protocol and uniform spirometers with regular calibration were used to ensure a reliable lung function measurement.

In this study, we chose Kriging interpolation for exposure estimation. One study has demonstrated better estimation performances of Kriging than the inverse distance weighting (IDW) or the nearest monitor estimation method on estimating PM_10_, NO_2_, CO, O_3_, and SO_2_^25^. The major limitation of kriging is that it does not consider environmental factors, such as land use and meteorological conditions^26^. The land-use regression (LUR) model may solve above problem and provide high local resolution. However, the generalizability of LUR is often concerned^27^. Hence, this method was not chosen since our participants were enrolled from four different geographic areas. In addition, satellite-based remote sensing approach^28^ was not feasible due to the high missing rates of the satellite-based aerosol optical depth measurements in Northern Taiwan due to meteorological conditions and cloud contamination. Finally, the accuracy of Kriging interpolation for PM_2.5_ and PM_10_ (10-fold cross-validated R^2^ 0.61 and 0.50, respectively) in this study was not inferior to land-use regression estimation adopted from Wu *et al*.^29^ (0.57 for PM_2.5_ and 0.50 for PM_2.5–10_). In conclusion, we considered Kriging interpolation an acceptable choice among current available methods.

Nonetheless, this study has some limitations that should be noted. First, given the cross-sectional design, caution has to be taken in determining a causal relationship. Further longitudinal studies are warranted to confirm the results. Second, due to the lack of chemical analysis of PM, only the health effect by mass concentration can be evaluated. The specific contribution of each source to the observed effect cannot be concluded. In the Taipei area, the main source of PM_10_ is soil dust (34%), followed by vehicle emissions (25%) and secondary aerosols (24%). In contrast, the main source of PM_2.5_ is from vehicle emissions (33%), followed by industrial emissions (23%), secondary aerosols (22%), and soil dust (20%)^29^. A study in Chiayi reveals that the main source of PM_2.5_ is secondary aerosols (33%), followed by traffic exhaust (16%), the petrochemical industry (9%), and agricultural burning (8%)^30^. Another study in Kaohsiung City demonstrates that the main source of PM_2.5_ is traffic exhaust (18–54%), followed by secondary aerosols (30–45%) and agricultural burning (13–17%)^31^. Information regarding the geographic distribution of PM_2.5–10_ is limited. Long-term monitoring of particle composition in the epidemiologic survey areas should be done in the future to examine specific sources with related health effects. Lastly, the range of ozone exposure among the study subjects is quite narrow. This reduces the detectability of a exposure-response relationship, even though evidence has shown the negative effect of ozone on lung function^6,32,33^.

In conclusion, this study demonstrates that long-term exposure to both ambient PM_2.5_ and PM_2.5–10_ at residential sites is associated with reduced lung function among the elderly (aged ≥65 years). The observed exposure-response relationship of PM_2.5_ on respiratory function in this study was larger than in previous one conducted in a lower polluted country. Moreover, there are different effects on lung function by PM_2.5_ and PM_2.5–10_, the former with stronger effect on vital capacity and the later on airway parameters. These results emphasize the constant need for stricter control of air pollution, including both fine and coarse PM, and for more studies on long-term health outcomes, especially among the vulnerable population.

## Supplementary information

Supplementary information
