# Supplementary material for: The effects of fine and coarse particulate matter on lung function among the elderly
Source: Sci Rep. 2019 Oct 15;9:14790. doi: 10.1038/s41598-019-51307-5 (PMC6794286; doi:10.1038/s41598-019-51307-5)
Supplement: Supplementary file 1 — Supplementary information [file 41598_2019_51307_MOESM1_ESM.docx]

**Supplementary Information.**

**Title:**

**The effects of fine and coarse particulate matter on lung function among the elderly**

**Authors:**

Chi-Hsien Chen^1,2^, Chih-Da Wu^3^, Hung-Che Chiang^4^, Dachen Chu^5,6,7,8^, Kang-Yun Lee^9,10^, Wen-Yi Lin^11^, Jih-I Yeh^12^, Kun-Wei Tsai^13^, and Yue-Liang Leon Guo^2,4,14^

**Authors’ details**

^1^Department of Environmental and Occupational Medicine, National Taiwan University Hospital Hsin-Chu Branch, Hsinchu, Taiwan.

^2^Department of Environmental and Occupational Medicine, National Taiwan University (NTU) College of medicine and NTU Hospital, Taipei, Taiwan

^3^Department of Geomatics, National Cheng Kung University, Tainan, Taiwan

^4^National Institute of Environmental Health Sciences, National Health Research Institutes, Miaoli County, Taiwan

^5^School of Medicine, National Yang-Ming University, Taipei, Taiwan

^6^Institute of Public Health and Community Medicine Research Center, National Yang-Ming University, Taipei, Taiwan

^7^Department of Health Care Management, National Taipei University of Nursing and Health Sciences, Taipei, Taiwan

^8^Department of Neurosurgery, Taipei City Hospital, Taipei, Taiwan

^9^Division of Pulmonary Medicine, Department of Internal Medicine, Shuang Ho Hospital, Taipei Medical University, New Taipei City, Taiwan

^10^Department of Internal Medicine, School of Medicine, College of Medicine, Taipei Medical University, Taipei, Taiwan

^11^Department of Occupational Medicine, Health Management Center, Kaohsiung Municipal Siaogang Hospital, Kaohsiung Medical University

^12^Department of Family Medicine, Hualien Tzu-Chi General Hospital

^13^Division of Geriatrics, Dalin Tzu-Chi Hospital, Buddhist Tzu Chi Medical Foundation

^14^Institute of Occupational Medicine and Industrial Hygiene, National Taiwan University, Taipei, Taiwan

**Corresponding Author:**

Prof. Yue-Liang Leon Guo

Department of Environmental and Occupational Medicine
College of Medicine

National Taiwan University and

National Taiwan University Hospital

Rm 339, 17 Syujhou Road, Taipei 100, Taiwan

Phone: +886-2-3322-8216

Fax: +886-2-3322-8214

E-mail: [leonguo@ntu.edu.tw](mailto:leonguo@ntu.edu.tw)

**Supplementary Table S1.** The association between each ambient air pollutant and pulmonary outcomes in a single-pollutant model in the subgroup without obstructive lung diseases (n=1197)

|  | FVC | FEV_1_ | FEV_1_/FVC |  |
| --- | --- | --- | --- | --- |
| PM_2.5_ | **-108.61 (23.33)****** | **-74.76 (19.03)****** | 0.006 (0.004) |  |
| PM_2.5-10_ | **-101.83 (26.27)****** | **-89.57 (21.35)****** | -0.001 (0.004) |  |
| NO_2_ | -1.35 (29.45) | 12.04 (23.96) | 0.003 (0.005) |  |
| CO | 28.01 (29.02) | 32.02 (23.61) | 0.002 (0.005) |  |
| O_3_ | -8.70 (26.76) | -17.94 (21.77) | -0.003 (0.004) |  |
| SO_2_ | **-15.02 (4.32)***** | **-9.09 (3.45)**** | 0.001 (0.001) |  |
|  |  |  |  |  |
|  | MMEF | FEF_25%_ | FEF_50%_ |  |
| PM_2.5_ | -32.17 (37.23) | **-423.91 (67.33)****** | -94.68 (48.89) |  |
| PM_2.5-10_ | **-107.29 (41.71)*** | **-486.63 (75.56)****** | **-171.88 (54.76)**** |  |
| NO_2_ | 31.12 (46.59) | 67.97 (85.62) | -1.15 (61.26) |  |
| CO | 36.70 (45.92) | **217.55 (84.19)**** | 20.95 (60.39) |  |
| O_3_ | -44.79 (42.33) | -90.21 (77.79) | -41.83 (55.66) |  |
| SO_2_ | 0.72 (6.73) | **-77.66 (12.17)****** | -9.35 (8..85) |  |
| *p<0.05; **p<0.01; ***p<0.001; ****p<0.0001 | | | | |
| Models were adjusted by age, gender, body height, body weight, education, current tobacco smoke, former tobacco smoke, cumulative pack-year of smoking, season of lung function test | | | | |
| Regression coefficients and standard errors were estimated for an interquartile range increase in each pollutant. | | | | |

**Supplementary Table S2.** The interaction between fine and coarse particulate matters on lung function

|  | FVC | | FEV_1_ | | FEV_1_/FVC | |
| --- | --- | --- | --- | --- | --- | --- |
|  | Coeff. | p value | Coeff. | p value | Coeff. | p value |
| PM_2.5_ (high vs low) | -96.49 | 0.0007 | -39.89 | 0.087 | 0.013 | 0.0072 |
| PM_2.5-10_ (high vs low) | -59.97 | 0.034 | -90.43 | 0.0001 | -0.015 | 0.0019 |
| PM_2.5_*PM_2.5-10_ |  | 0.872 |  | 0.489 |  | 0.065 |
|  | MMEF | | FEF_25_ | | FEF_50_ | |
|  | Coeff. | p value | Coeff. | p value | Coeff. | p value |
| PM_2.5_ (high vs low) | 54.99 | 0.219 | -218.91 | 0.008 | 14.68 | 0.803 |
| PM_2.5-10_ (high vs low) | -176.35 | <0.0001 | -471.19 | <0.0001 | -234.12 | <0.0001 |
| PM_2.5_*PM_2.5-10_ |  | 0.089 |  | 0.439 |  | 0.064 |

Median of the concentration of each PM was used to stratify subjects into high and low exposure groups.

Models were adjusted by age, gender, body height, body weight, physician-diagnosed asthma and physician-diagnosed COPD, education, current tobacco smoke, former tobacco smoke, cumulative pack-year of smoking, and season of lung function test.

**Supplementary Table S3.** The association between each ambient particulate air pollutant and lung function indices in a two-pollutant model in the subgroup without obstructive lung diseases (n=1197)

|  | FVC | FEV_1_ | FEV_1_/FVC | MMEF | FEF_25%_ | FEF_50%_ | |
| --- | --- | --- | --- | --- | --- | --- | --- |
| PM_2.5_ |  |  |  |  |  |  | |
| with PM_2.5-10_ | **-100.27*** | -30.43 | **0.018**** | **122.75*** | -214.66 | 76.92 | |
| with NO_2_ | **-108.79****** | **-75.35***** | 0.006 | -33.37 | **-427.28****** | -94.84 | |
| with CO | **-107.80****** | **-72.45***** | 0.006 | -28.02 | **-405.85****** | -94.46 | |
| with O_3_ | **-108.68****** | **-73.40***** | 0.006 | -29.27 | **-420.28****** | -92.38 | |
| with SO_2_ | **-114.74**** | **-93.68**** | 0.003 | -94.64 | **-224.49*** | -144.07 | |
|  |  |  |  |  |  |  | |
| PM_2.5-10_ |  |  |  |  |  |  | |
| with PM_2.5_ | -11.86 | -62.24 | **--0.018*** | **-217.54**** | **-293.83*** | **-240.97**** | |
| with NO_2_ | **-108.54****** | **-92.42****** | -0.0006 | **-106.82*** | **-501.48****** | **-182.97**** | |
| with CO | **-111.73***** | **-94.70***** | -0.0007 | **-114.24*** | **-493.34***** | **-201.37***** | |
| with O_3_ | **-112.06***** | **-94.63****** | -0.0003 | **-104.40*** | **-517.87****** | **-178.73**** | |
| with SO2 | **-71.69*** | **-86.48**** | -0.008 | **-169.37**** | **-309.39**** | **-211.83**** | |
|  |  |  |  |  |  |  | |
| SO_2_ |  |  |  |  |  |  | |
| with PM_2.5_ | 1.40 | 4.32 | 0.0007 | 14.26 | **-45.53*** | 11.27 | |
| with PM_2.5-10_ | -8.19 | -0.85 | **0.002*** | **16.87*** | **-48.15**** | 10.85 | |
| with NO_2_ | **-18.21***** | **-11.97**** | 0.001 | -1.45 | **-99.69****** | -11.30 | |
| with CO | **-16.47***** | **-10.42**** | 0.001 | -0.36 | **-87.32****** | -10.35 | |
| with O_3_ | **-16.79***** | **-10.79**** | 0.001 | -1.44 | **-89.05****** | -12.25 | |
| *p<0.05; **p<0.01; ***p<0.001; ****p<0.0001 | | | | | | |  |
| Models were adjusted by age, gender, body height, body weight, education, current tobacco smoke, former tobacco smoke, cumulative pack-year of smoking, season of lung function test, and co-pollutants. | | | | | | |  |
| Regression coefficients were estimated for an interquartile range increase in each pollutant. | | | | | | |  |
